# Scientific Items

**Published:** 1887-12

**Authors:** 


### SCIENTIFIC ITEMS.

**Electrical Ice Cream Poison.**—Dr. George S. Hull, of Chambersburg, Pennsylvania, advances the theory that ice cream poisoning is due to chemical action which takes place in the ice cream freezer, and which dissolves the zinc. He demonstrated his theory by means of a galvanometer.

In conducting the experiments, the doctor connected the zinc, paddle, and tin can by means of a copper wire with a moderately sensitive galvanometer introduced in the circuit. He first experimented with pure cream, which deflected the galvanometer seven degrees at the freezing point, thus showing that some slight solution was taking place, and proving that if pure cream caused a deflection of seven degrees, the other ingredients of ice cream would probably cause more, and in such cases sufficient zinc would be dissolved in the cream as ordinarily made as would make it decidedly dangerous as a delicacy. The deflections caused by various other substances used were: Half cream and milk, 25 degrees; pure milk, 45 degrees; one-half cream, half milk, sugar and vanilla flavor, 58; the same mixture with corn starch, 44; the same mixture with eggs, 80.—*Scientific American.*

**Vest Button Photography.**—The process of iustantaneous photographing is rapidly becoming an evil. We hear talk already about specialists in photography for instantaneous pictures since the "Detective Camera," as it is called, was put upon the market. The box is so small that it can be carried anywhere without the slighest inconvenience, and as the little lens at the bottom is always ready f r use, an instantaneous picture can be taken at any desired moment. There was some misgiving at first entertained about the value of these cameras, but we have recently seen some wonderful work produced by them. In the camera is a gelatine plate which can be turned six times, so that six photographs can be taken one after the other, and these pictures are so sharply defined that they can be enlarged tenfold. The inventor, Mr. Stirn, of New York, is a German by birth, and his brother, Mr. R. Stirn, of Bremen, sells the apparatus for about seven dollars (thirty marks), with a complete outfit. No operator is required to fit the camera and lens correctly in position for the party to be photographed. All that is requisite is to pull a string and the photograph is at once taken. With another pull the plate is made ready for another picture. We are told that the most prominent artists carry this detective camera with them at all times—A. Von Werner, Gussow, Thumann, O. Lessing, and others. This they do from a strictly professional point of view, and not as amateurs or dillettanti. In Germany, Herr Stein recently produced before a photographic society enlargements to a size of forty centimeters from such pictures, and all were remarkably distinct and well defined. These plates can also be taken at different distances and always sharply outlined. Young men take their lady friends on promenade to be unconsciously photographed. People, young and old, who have never entered an artist's studio or a photograph gallery will be astonished to see their pictures freely circulated. Most of all, it is to be feared that the legitimate business of the photographer will be injured by these cameras. Any possible mania or desire for photos. can soon be gratified at trifling expense and after a term of practice by means of this invention. Photographs can soon be so multiplied as to become a positive nuisance, and from the various considerations that enter into the matter, it does not seem so very easy to answer our query—"What next?"—*American Lithographer.*

**Arctic Cold.**—A person who has never been in the polar regions can probably have no idea of what cold really is; but by reading the terrible experiences of arctic travelers in that icy region some notion can be formed of the extreme cold that prevails there. When we have the temperature down to zero out of doors we think it bitterly cold, and if our houses were not so warm as, at least, 60 degrees above zero, we should begin to talk of freezing to death. Think, then, of living where the thermometer goes down to 45 degrees *below* zero in the house in spite of the stove. Of course, in such a case the fur garments are piled on until a man looks like a great bundle of skins. Dr. Moss, of the English polar expedition of 1875 and 1876, among other odd things, tells of the effect of cold on a wax candle which he burned there. The temperature was 35 degrees below zero, and the doctor must have been considerably discouraged when, upon looking at his candle, he discovered that the flame had all it could do to keep warm. It was so cold that the flame could not melt all the wax of the candle, but was forced to eat its way down the candle, leaving a sort of skeleton of the candle standing. There was heat enough, however, to melt oddly shaped holes in the thin walls of wax, and the result was a beautiful lace-like cylinder of white, with a tongue of yellow flame burning inside it and sending out into darkness many streaks of light. This is not only a curious effect of extreme cold, but it shows how difficult it must be to find anything like warmth in a place where even fire itself almost gets cold. The wonder is that any man can have the courage to willingly return to such a bitter region after having once got safely away from it, and yet the truth is that the spirit of adventure is so strong in some men that it is the very hardship and danger which attract them.—*Scientific American*, August 13, 1887.

**Home-made Ice.**—Take a cylindrical earthen vessel and pour $3\frac{1}{2}$ ounces of commercial sulphuric acid and $1\frac{3}{4}$ ounces of water into it and then add 1 ounce of powdered sulphate of soda In the center of this mixture, place a small vessel, and, if possible, revolve the whole with a gentle motion. In a few minutes, the water in the small vessel will be converted into ice. The same mixture can be used a second or third time for making a block of ice. The operation should, if possible, be performed in a cool place, in a cellar, for example.—*La Science en Famille.*

